# Supplementary material for: Enhancing Clinical Classifier Robustness with Deep Representation Learning Based OOD Detection on DNA Methylation Data
Source: ArXiv. 2026 Oct 26:arXiv:2610.36344v1. Preprint. [Version 1] (PMC13734156)
Supplement: Supplementary file 1 [file NIHPP2610.36344v1-supplement-1.pdf]

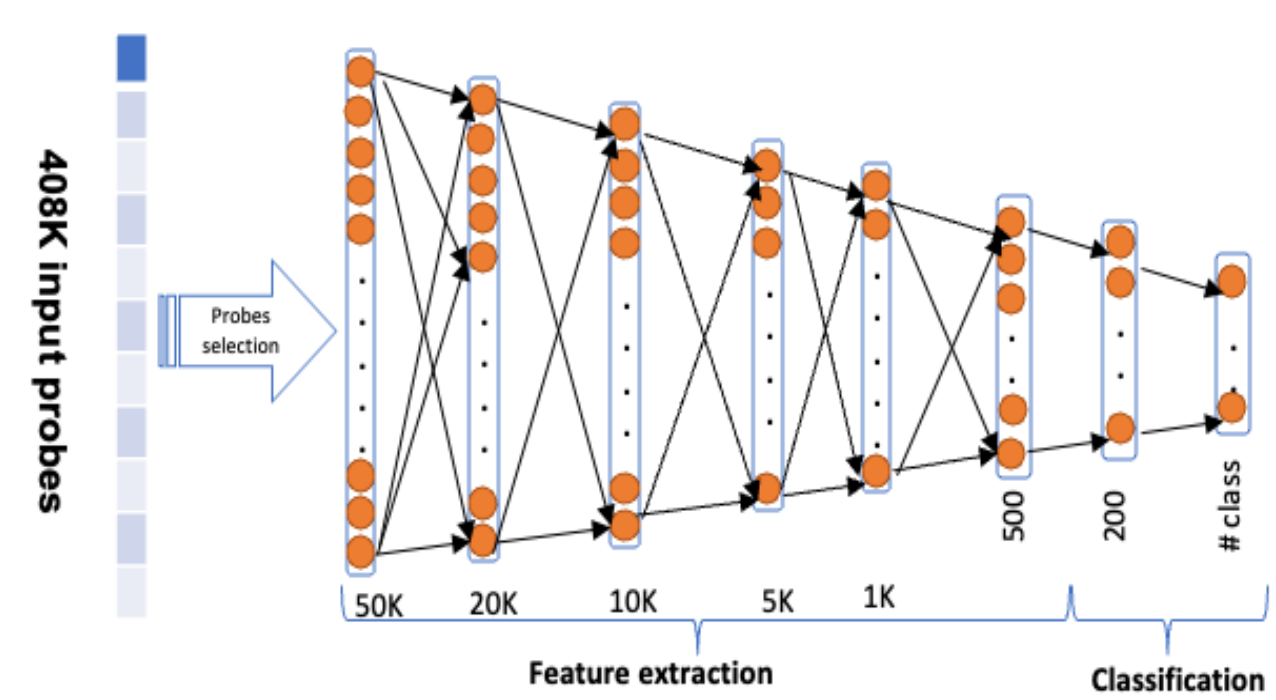

Architectures:

SARC(IND) and BT(OOD) :

50,000 → 20,000 → 10,000 →5,000 → 1,000→500→ 200→ 58

408,768 → 3,000 → 2000 →1,500 → 1,000 →500 → 200 → 58

BT(IND) and SARC(OOD) :

50,000 → 20,000 → 10,000 →5,000 → 1,000→500→ 200→ 84

408,768 → 3,000 → 2000 →1,500 → 1,000 →500 → 200 → 84

Supplementary Fig. 1. The Deep Neural Networks (DNN) architectures for both experimental setups: the DNN architecture for 50K input probes from 408K, and the architectures for 408K input probes.

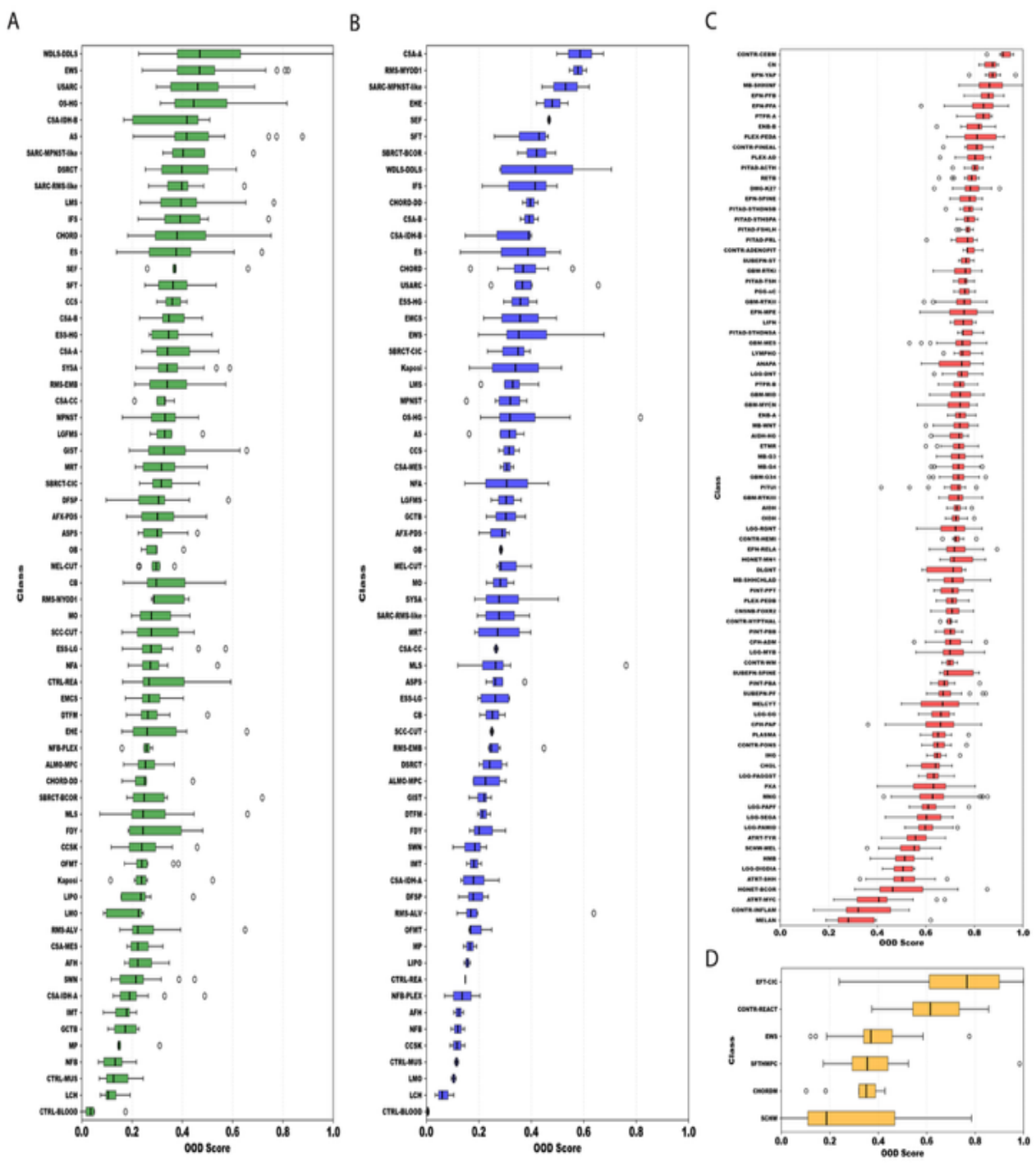

Supplementary Fig. 2. Class-wise OOD Score Distribution (Ordered by Median) of training and testing examples for SARC(IND) and BT(OOD): (A) and (B) show the class-specific box plots for SARC training (IND) and testing (IND) samples, respectively. (C) Class-wise box plots for BT (OOD) examples, which show a higher OOD score compared to IND samples, and (D) shows the box plots for IND samples from the Brain Tumor datasets.

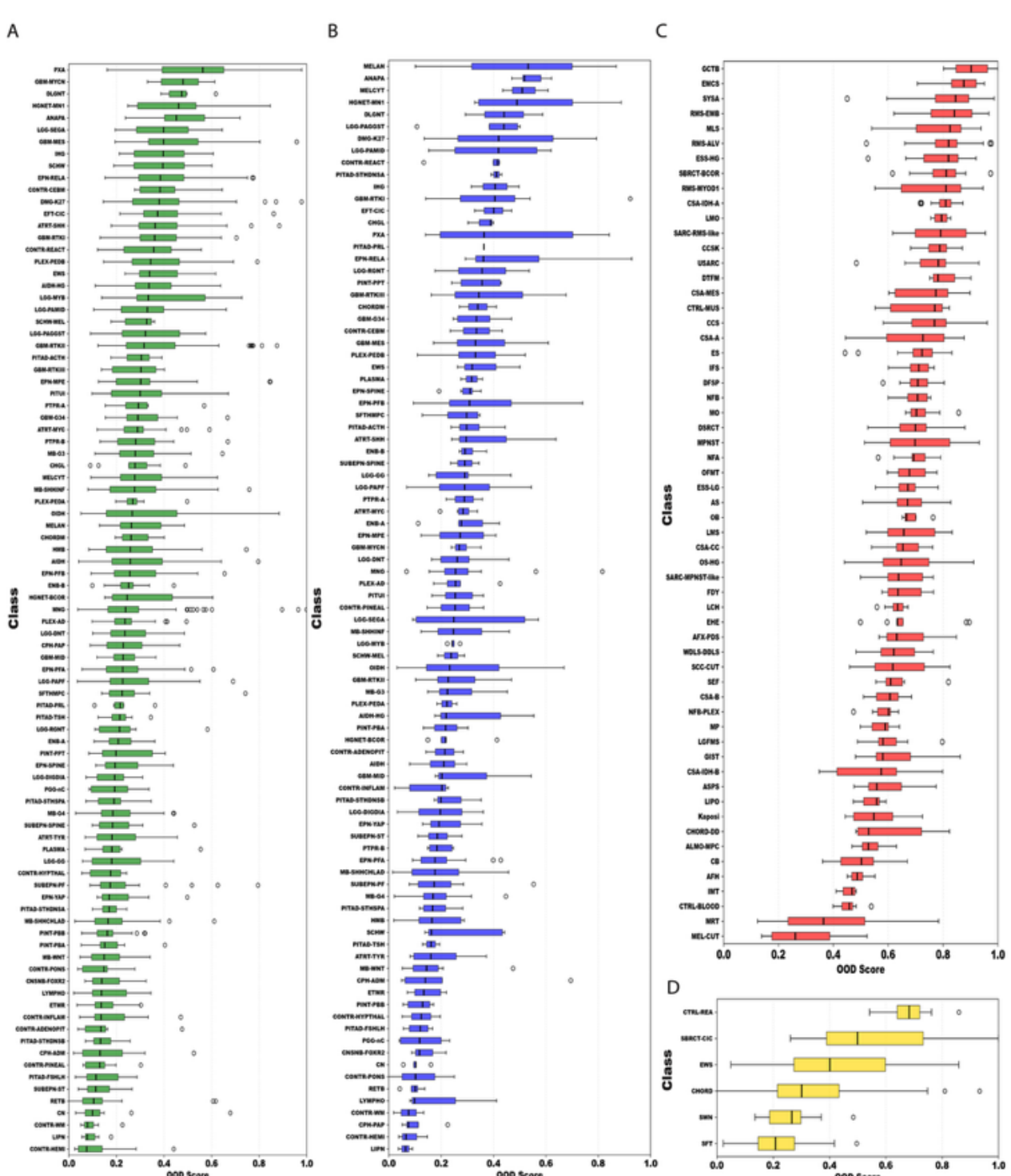

Supplementary Fig. 3. Class-wise OOD Score Distribution (Ordered by Median) of training and testing examples for Brain Tumor (BT)(IND) and SARCOMA(OOD): (A) and (B) show the class-specific box plots for BT training (IND) and testing (IND) samples, respectively. (C) Class-wise box plots for SARC (OOD) examples, which show a higher OOD score compared to IND samples, and (D) IND samples from the SARCOMA datasets.
